# SARS-COV-2 infection outcomes in patients with congenital generalized lipodystrophy

**DOI:** 10.1186/s13098-021-00680-1

**Published:** 2021-06-13

**Authors:** Mayara Ponte Madeira, Erika Bastos Lima Freire, Virginia Oliveira Fernandes, Grayce Ellen da Cruz Paiva Lima, Ivana da Ponte Melo, Ana Paula Dias Rangel Montenegro, José Ednésio da Cruz Freire, Caroline de Fátima Aquino Moreira-Nunes, Raquel Carvalho Montenegro, Jeová Keny Baima Colares, Renan Magalhães Montenegro Junior, Brazilian Group for the Study of Inherited and Acquired Lipodystrophies (BRAZLIPO)

**Affiliations:** 1 Clinical Research Unit, Walter Cantidio University Hospital, Federal University of Ceará, Bloco das Ilhas – 1º Andar, Fortaleza, CE 60430-270 Brazil; 2 Department of Clinical Medicine, Federal University of Ceará, Fortaleza, CE Brazil; 3 Department of Community Health, Federal University of Ceará, Fortaleza, CE Brazil; 4 Drug Research and Development Center (NPDM), Federal University of Ceará, Fortaleza, CE Brazil; 5 PostGraduate Program in Medical Sciences, University of Fortaleza, Fortaleza, CE Brazil; 6 Faculdade de Medicina, Universidade Federal do Ceará, Rua Professor Costa Mendes 1608, Fortaleza, CE 60416-200 Brazil

**Keywords:** SARS-COV-2, COVID-19, Congenital generalized lipodystrophy, Lipodystrophy, Diabetes, Insulin resistance

## Abstract

**Background:**

A new strain of human coronavirus (HCoV) spread rapidly around the world. Diabetes and obesity are associated with a worse prognosis in these patients. Congenital Generalized Lipodystrophy (CGL) patients generally have poorly controlled diabetes and require extremely high doses of insulin. There is no documentation in the literature of cases of COVID in CGL patients. Thus, we aimed to evaluate the prevalence of SARS-CoV-2 infection in CGL patients**,** and the association of their clinical and metabolic characteristics and outcomes.

**Methods:**

This is a cross-sectional study carried out between July and October 2020. Clinical data collected were respiratory or other flu-like symptoms, need of hospitalization in the last three months, CGL comorbidities, and medications in use. Cholesterol, triglycerides, glycohemoglobin A1c levels, anti-SARS-CoV-2 antibodies and nasopharyngeal swab for RT-qPCR were also obtained in all CGL patients. Mann-Whitney U test was used to analyze the characteristics of the participants, verifying the non-adherence of the data to the Gaussian distribution. In investigating the association between categorical variables, we used Pearson's chi-square test and Fisher's exact test. A significance level of 5% was adopted.

**Results:**

Twenty-two CGL patients were assessed. Eight subjects (36.4%) had reactive anti-SARS-CoV-2 antibodies. Only one of these, also presented detectable RT-qPCR. Five individuals (62.5%) were women, median age of 13.5 years (1 to 37). Symptoms like fever, malaise, nausea, diarrhea and chest pain were present, and all asymptomatic patients were children. All subjects had inadequate metabolic control, with no difference between groups. Among positive individuals there was no difference between those with *AGPAT2* (75%) and *BSCL2* gene mutations (25%) (*p *> 0.05). No patient needed hospitalization or died.

**Conclusions:**

We described a high prevalence of SARS-CoV-2 infection in CGL patients with a good outcome in all of them. These findings suggest that at least young CGL patients infected by SARS-COV-2 are not at higher risk of poor outcome, despite known severe metabolic comorbidities.

## Background

In December 2019, a new strain of human coronavirus (HCoV) emerged in Wuhan (China). It causes the severe acute respiratory syndrome coronavirus 2 (SARS-CoV-2), which spread rapidly around the world [1–3]. From March 2020, the authorities of the state of Ceará, located in northeast region of Brazil, established a series of restrictions to reduce the spread of the infection. These measures culminated in an almost complete blockade of the state [4]. In June 2020, Brazil was the second most affected country around the world [5].

Diabetes *mellitus* (DM) and obesity are associated with worst prognosis in patients infected with SARS-CoV-2 [6–15]. It is still uncertain whether this susceptibility to the disease severity is particularly high or reflects the increased risk caused by these conditions. Older age, comorbidities such as hypertension, cardiovascular disease, obesity, and a pro-inflammatory and pro-coagulative state probably contribute to the risk of worst outcomes [8, 16–21].

Congenital generalized lipodystrophy (CGL) is a rare disease caused by autosomal recessive inheritance [22]. There are approximately 500 patients described worldwide, with more than 100 cases described in Brazil [23–27]. CGL patients have total or almost total loss of body fat. This abnormality causes ectopic fat accumulation in organs, such as the liver and muscles. Patients evolve with hypertriglyceridemia, severe insulin resistance and poorly controlled diabetes, that usually require extremely high doses of insulin [28].

CGL patients have a greater predisposition to severe infectious diseases which is one of the main causes of mortality among this population [29]**.** There is no documentation in the literature of cases of SARS-CoV-2 in CGL patients.

Our study aims to evaluate the prevalence of SARS-CoV-2 infection in CGL patients**,** and the association of their clinical and metabolic characteristics and outcomes.

## Methods

### Study design and participants

This is a cross-sectional study carried out between July and October 2020. Patients with CGL diagnosis enrolled at the Endocrinology Service at the University Hospital Walter Cantídio were invited by telephone to attend for clinical reassessment and participation in the study. This hospital, which is head office of the Brazilian Group for the Study of Inherited and Acquired Lipodystrophies (BRAZLIPO), is a reference on the care lipodystrophy patients. The main inclusion criteria was the CGL clinical diagnosis (total or near total absence of body fat associated with muscle hypertrophy present at birth or soon thereafter, phlebomegaly, acromegaloid facies, increased liver volume, insulin resistance and hypertriglyceridemia) [30]. Currently, 24 CGL patients are followed up. Those who couldn't attend to regular follow up were excluded. The present study was approved by the ethics committee of University Hospital Walter Cantídio (protocol number: 4.255.817). Written informed consent was obtained from all adult participants and written parental consent was obtained for all participants aged under 18.

### Data collection

The patients attended an interview and answered a structured questionnaire with clinical and sociodemographic data, smoking habit, presence of DM, dyslipidemia or other CGL comorbidities, and medications in use. They also were asked about the presence of respiratory or other flu-like symptoms and/or need of hospitalization in the last three months.

Blood glucose, cholesterol, and triglyceride levels were obtained after overnight fast and determined according to standard methods using automated equipment. Glycohemoglobin A1c (A1c) values were determined by ion exchange high-performance liquid chromatography (HPLC).

Blood samples for detection of anti-SARS-CoV-2 antibodies, including IgG (Elecsys® Anti-SARS-CoV-2—Roche Diagnostics) were collected in all patients. Elecsys® Anti-SARS-CoV-2—Roche Diagnostics is immunoassay for the in-vitro qualitative detection of COVID antibodies in human serum and plasma and has 99.81% specificity [31].

The patients also were referred to a private room by a trained health professional with personal protective equipment to collect the nasopharyngeal swab. Presence of viral RNA was determined in all samples by specific real-time polymerase chain reaction (RT-PCR) assay through the Berlin Protocol—Molecular Kit SARS-CoV2 Biomanguinhos (E / RP). Detectable result indicated the presence of the two RNA regions of the SARS-CoV-2 virus [32].

### Statistical analysis

In the numerical variables, the data were presented in median (minimum–maximum). In categorical variables, data were exposed in terms of frequency and prevalence rate. Mann-Whitney U test was used to analyze the characteristics of the participants, verifying the non-adherence of the data to the Gaussian distribution. In investigating the association between categorical variables, we used Pearson's chi-square test and Fisher's exact test. A significance level of 5% was adopted. Statistical analyzes were performed using the statistical program JAMOVI and Microsoft Excel 2016.

## Results

### Sample description

Of the 24 patients under follow-up, 22 CGL patients from 17 different families were included. Thirteen subjects (59%) were women. Median age was 20 years, ranging from 1 to 42 years. Almost all families lived in the state of Ceará. Eight patients (36%) came from the capital (Fortaleza) or metropolitan region and fourteen individuals (64%) from other cities of Ceará. Only one patient was from state of Rio Grande do Norte. No adult referred alcohol ingestion and only one had smoking habit.

Regarding comorbidities, 21 (95%) patients presented hypertriglyceridemia. Two of these (9.5%) were in fibrate use. Two patients (9%) had hypertension. Eighteen patients (81.8%) had DM with a disease duration of 10 years (0.1–21 years). All diabetics used metformin, except one patient because she was on dialysis. Twelve subjects (75%) were on insulin therapy, with a daily average dose of 1.63 IU/kg (0.4–7.25). Three non-diabetics were using metformin due to insulin resistance.

The molecular analysis identified *AGPAT2* gene mutations in 16 patients (72.8%) and *BSCL2* mutations in 6 patients (27.2%).

Clinical and genotypic characteristics of the 22 CGL patients are described in the Table 1.

**Table 1:** Clinical and genotypic characteristics of CGL patients.

Case Age/gender	Mutation	Comorbidities and chronic complications	Tests for SARS-CoV-2	A1C levels	HDL levels	No-HDL levels	TG levels	Medications in use
1 16, M	AGPAT2	DM, ↑TG, ↓ HDL, HS, nephropathy (albuminuria), peripheral neuropathy	Non-reactive	9.7	29	120	384	Insulin (7.25), MTF
2 32, F	AGPAT2	DM, ↑TG, ↓ HDL, SAH, CAD, HS, CKD on dialysis, peripheral neuropathy	Reactive	6.6	23	91	125	Insulin (5.8), statine
3 37, F	AGPAT2	DM, ↑TG, ↓ HDL, HS, nephropathy (albuminuria), peripheral neuropathy	Reactive	9.9	22	56	142	Insulin (1.71), MTF, statine
4 42, F	AGPAT2	DM, ↑TG, ↓ HDL, SAH, HS, retinopathy	Non-reactive	5.6	25	96	191	Insulin (1), MTF, statine
5 24, F	AGPAT2	DM, ↑TG, ↓ HDL, HS	Non-reactive	5.6	24	133	664	MTF, fibrate
6 32, F	AGPAT2	DM, ↑TG, ↓ HDL, HS, nephropathy (albuminuria), peripheral neuropathy, retinopathy	Non-reactive	11.2	31	200	634	Insulin (1.55), MTF, fibrate, statine
7 7, F	AGPAT2	↑TG, ↓ HDL, HS	Reactive	5.2	39	109	49	None
8 33, M	AGPAT2	DM, ↑TG, ↓ HDL, HS, nephropathy (albuminuria), retinopathy	Reactive	11.2	40	110	114	Insulin (1.32), MTF
9 7, M	AGPAT2	↑TG, ↓ HDL, HS	Non-reactive	5.3	26	118	268	MTF
10 10, M	AGPAT2	↑TG, ↓ HDL	Non-reactive	5	47	124	95	MTF
11 1, M	AGPAT2	DM, ↑TG, ↓ HDL	Reactive	5.5	24	79	144	None
12 11, F	AGPAT2	DM, ↑TG, ↓ HDL, HS	Reactive	8.6	41	172	427	MTF
13 13, F	AGPAT2	DM, ↑TG, ↓ HDL, HS, peripheral neuropathy	Non-reactive	6.8	24	123	436	MTF
14 20, M	AGPAT2	DM, ↑ TG, ↓ HDL, HS, peripheral neuropathy	Non-reactive	9.9	34	153	262	MTF, statine
15 26, F	AGPAT2	DM, ↑TG, ↓ HDL, HS, nephropathy (albuminuria), retinopathy	Non-reactive	11.4	37	198	635	Insulin (1.96), MTF
16 29, F	AGPAT2	DM, ↑TG, ↓ HDL, HS, nephropathy (albuminuria), peripheral neuropathy, retinopathy	Non-reactive	5.5	20	121	891	Insulin (3.24), MTF, fibrate, statine
17 41, M	AGPAT2	DM, ↑TG, ↓ HDL, HS, retinopathy	Non-reactive	7.3	26	162	524	Insulin (1.41), MTF
18 9, M	BSCL2	↑TG, ↓ HDL	Reactive	5	27	129	289	MTF
19 12, F	BSCL2	DM, ↑TG, ↓ HDL, HS, nephropathy (albuminuria), peripheral neuropathy	Non-reactive	8.2	33	127	526	MTF
20 16, F	BSCL2	DM, ↑TG, ↓ HDL, HS, peripheral neuropathy	Reactive	11	33	138	141	Insulin (2.58), MTF
21 21, M	BSCL2	DM, ↑TG, ↓ HDL, HS, nephropathy (albuminuria), peripheral neuropathy	Non-reactive	10.4	45	146	139	Insulin (1.53), MTF
22 35, F	BSCL2	DM, ↓ HDL, HS	Non-reactive	5.6	29	83	80	Insulin (0.39), MTF, statine

*CAD* coronary artery disease, *CKD* chronic kidney disease, *DM* diabetes mellitus, *F* female, *HDL* high-density lipoprotein, *HS* hepatic steatosis, *M* male, *MTF* metformin, *SAH* systemic arterial hypertension, *TG* triglycerides

The numbers in parentheses are the number of units of insulin per kilogram of weight

### SARS-CoV-2 infection outcomes in CGL patients

**Fig. 1:**
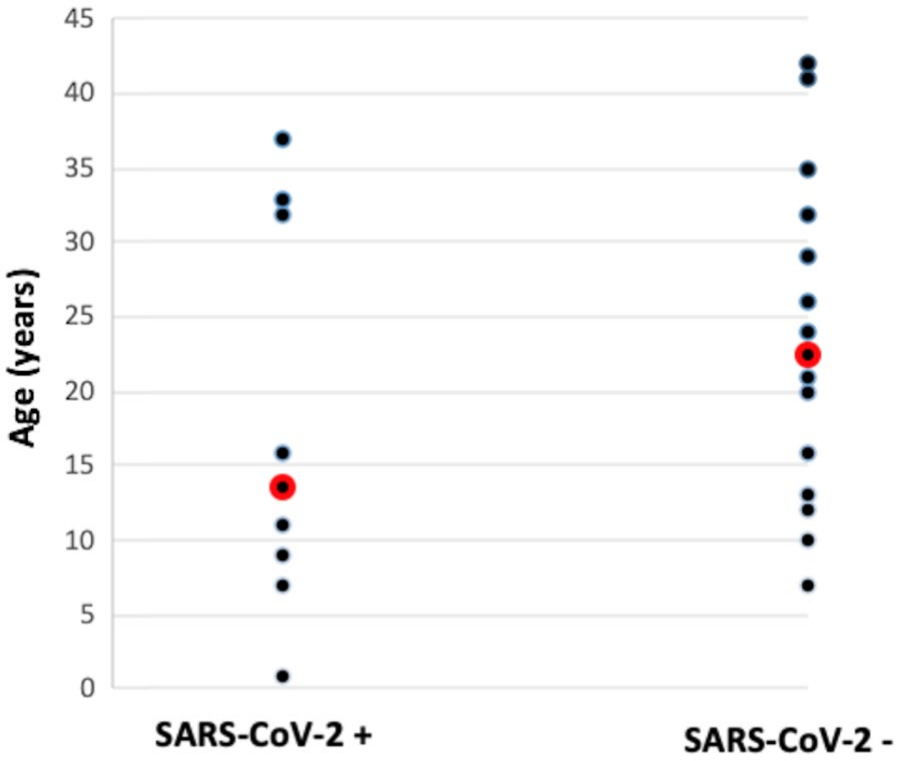
Age range and median age (in red) of in CGL patients with reactive (SARS-COV-2+) and non-reactive serologic tests for SARS-CoV-2 infection (SARS-COV-2−) (*n* = 22, *p* = 0.355)

Most patients presented non-reactive serologic tests for SARS-CoV-2 infection (SARS-COV-2−), but eight individuals (36.4%) had reactive results (SARS-COV-2+) (Fig. 1). Only one of them also presented detectable RT-qPCR nasopharyngeal swab. Five SARS-COV-2+ (62.5%) were women with median age 13.5 years (1 to 37) (Fig. 1). Four of these (50%) lived in the capital of Ceará.

Among SARS-COV-2+ patients there was no difference in clinical or metabolic profile between those with *AGPAT2* (75%) and *BSCL2* gene mutations (25%) (*p *> 0.05).

In this group all adults presented symptoms like fever, malaise, nausea, diarrhea or chest pain in the last 3 months before the visit, and all asymptomatic patients were children. We also observed symptoms among SARS-COV-2—patients without difference between groups (Table 2).

**Table 2:** Clinical characteristics and SARS-CoV-2 infection outcomes of CGL subjects with reactive (SARS-CoV-2+) and non-reactive (SARS-CoV-2) serologic tests

	SARS-CoV-2+	SARS-CoV-2-	*p*
Patients, n	8 (36.3%)	14 (63.6%)	
Female, n (%)	5 (62.5%)	8 (57.1%)	> 0.999
Age (years), median (min-max)	13.5 (1–37)	22.5 (7–42)	0.355
Under 18 years, n (%)	5 (62.5%)	5 (35.7%)	0.378
Mutation			
AGPAT2, n (%)	6 (75%)	10 (71.4%)	> 0.999
BSCL2, n (%)	2 (25%)	4 (28.6%)	> 0.999
Metabolic profile			
A1C median (min–max)	7.6 (5–11.2)	7.05 (5–11.4)	0.86
TC median (min–max)	149 (78–213)	158.5 (112–235)	0.159
HDL median (min–max)	30 (22–41)	29 (20–47)	0.909
Non-HDL median (min–max)	109.5 (56–172)	125.5 (83–200)	0.111
TG median (min–max)	141.5 (49–427)	410 (80–891)	0.023
Metabolic control			
A1C > 7.0%, n (%)**	4 (66%)	7 (58%)	> 0.999
No-HDL > 100, n (%)	5 (62.5%)	12 (85.7%)	0.5492
Hypertrygliceridemia*, n (%)	4 (50%)	10 (71.4%)	0.3864
Comorbidities			
DM, n (%)	6 (75%)	12 (85.7%)	0.602
Duration of DM (years), median (min–max)	6.5 (0.1-19)	11 (0.1-21)	0.501
Low HDL, n (%)	8 (100%)	14 (100%)	–
Hypertriglyceridemia, n (%)	8 (100%)	13 (92.8%)	> 0.999
Arterial hypertension, n (%)	1 (12.5%)	1 (7%)	> 0.999
Medicines in use			
Insulin, n (%)	4 (50%)	8 (57.2%)	> 0.999
Daily dose of insulin median, IU/kg/day	2.1 (1.3–5.8)	1.5 (0.4-7.2)	0.57
Metformin, n (%)	5 (62.5%)	13 (92.8%)	0.076
Fibrate, n (%)	0	2 (14.3%)	0.5152
Statine, n (%)	2 (25%)	5 (35.7%)	> 0.999
Clinical symptoms***			
Fever, n (%)	3 (37.5%)	3 (21.4%)	0.6244
Cough, n (%)	1 (12.5%)	2 (14.2%)	> 0.999
Sore throat, n (%)	1 (12.5%)	5 (35.7)	0.3512
Myalgia, n (%)	0	3 (21.4%)	0.2727
Fatigue, n (%)	0	0	–
Dyspnea, n (%)	1 (12.5%)	2 (14.2%)	> 0.999
Chest pain, n (%)	2 (25%)	1 (7.1%)	0.5273
Nausea, n (%)	3 (37.5%)	1 (7.1%)	0.1167
Diarrhea, n (%)	3 (37.5%)	3 (21.4%)	0.6244
No symptoms, n (%)	5 (62.5%)	6 (42.8%)	0.6594
Outcomes			
Hospitalization, n (%)	0		
Death, n (%) 0	0		

*A1C* glycohemoglobin A1c, *DM* diabetes mellitus, *HDL* high-density lipoprotein, *Non*-*HDL* non-high-density lipoprotein cholesterol, *TC* total cholesterol, *TG* triglycerides

The levels of triglycerides considered high, were classified according to the age group: in children between 0 and 9 years: > 75 mg/dL, between 10 and 19 years: > 90 mg/dL; and adults > 150 mg/dL [33]

**Among patients with diabetes ***Last 3 months

At the time of our evaluation, most patients in both SARS-COV-2+ and SARS-COV-2- had inadequate metabolic control, with no difference between groups (Table 2).

When comparing only CGL patients under 18 years there was no difference in genotype, clinical or metabolic characteristics between SARS-COV-2+ and SARS-COV-2− subjects (*p *> 0.05).

## Discussion

This is the first study to evaluate the prevalence of SARS-CoV-2 infection in CGL patients and the relationship with its clinical and metabolic profile during the SARS-CoV-2 pandemic.

In the present study, we describe a high prevalence of SARS-CoV-2 infection in CGL patients. This was observed in a predominantly young group, ranging from 1 to 37 years (median 13.5 years). A half of the subjects lived in the capital and metropolitan region of Ceará. Most patients had hypertriglyceridemia and DM, and 72.7% harbor *AGPAT2* gene mutations (Subtype 1 CGL).

At the time of our study, 2,65,680 COVID-19 cases had been confirmed in Ceará (Brazil) and the SARS-CoV-2 seroprevalence in Fortaleza (Ceará) was 13.1% [34]. The peak of COVID-19 cases in Ceará occurred between May and June 2020 and the research was carried on later when the incidence was lower. In this context, a higher detection of reactive serologic tests in CGL patients (36.4%) and a lower detection on RT-PCR test was plausible. CDC recommended serologic tests for SARS-CoV-2 infection as an important tool for epidemiologic studies. Unlike RT-PCR or antigen methods that detect acutely infected persons, antibody tests is useful to determine a previous SARS-CoV-2 infection even in a asymptomatic person [35].

Serious forms of COVID-19 have been associated with advanced age and several comorbidities, such as DM, hypertension, obesity, and cardiovascular disease. Such conditions tend to be interrelated in a network of causality that impair their proper understanding [36, 37]. CGL is associated with severe metabolic complications, poorly controlled DM that required extremely high doses of insulin [28]. Thus, more serious COVID-19 manifestations would be expected in our series. Several mechanisms have been suggested to explain the increased severity of COVID-19 in DM patients, such as impaired glucose control, diabetes-related immune dysfunction by decreasing viral clearance, reduced neutrophil chemotaxis and presence of monocytes that express higher levels of pro-inflammatory cytokines. Another rationale is the presence of concomitant comorbidities with higher expression of Angiotensin Converting Enzyme 2 (ACE2)*.* The viral binding of SARS-CoV-2 with ACE2 receptors may cause its downregulation, resulting in dysfunction of the renin angiotensin aldosterone system (RAAS), with uncontrolled activity of angiotensin II, which can contribute to acute lung injury [38–40]. Despite contradictory evidence, the presence of DM seems to be associated with increased tissue expression of ACE2 [41]. In our series most CGL patients had DM (81%) but none of them presented a poor outcome, even SARS-CoV-2+ patients.

One hypothesis for the asymptomatic disease or mild presentation of the infection found in our group would be the reduction of body fat in these patients, a primary reservoir of SARS-CoV-2 viruses. Obesity has been related to a worse outcome and prognosis of COVID-19. Adipose tissue demonstrates high expression of ACE2 that SARS-CoV-2 need to enter host cells and which makes adipose tissue a prime reservoir for SARS-CoV-2 viruses, thus increasing the integral viral load [42].

CGL patients have reduced leptin levels, which act in the modulation of the inflammatory response, proliferation and function of T lymphocytes, phagocytosis by macrophages and neutrophil chemotaxis. Thus, leptin deficient patients are more susceptible to infectious diseases [43]. Therefore, it would be expected that these individuals had a higher prevalence of more severe forms of COVID-19 and a high risk of death.

However, the predominantly young age of our population may balance these findings. Systematic review of COVID-19 in children showed milder cases and a better prognosis than in adults [44]. One hypothesis would be that the immune system in children can be protected against SARS-CoV-2 because ACE2 enzyme is less mature at a younger age [44, 45].

Our patients have the two most common CGL forms, Subtype 1 (mutations in *AGPAT2 gene)* and Subtype 2 (mutations in *BSCL2 gene*). *AGPAT2* gene is involved in lipogenesis, while *BSCL2* is involved in the maturation of preadipocytes and adipocytes. Subtype 2 is considered a more severe form, however there was no difference in prevalence or outcome of SARS-CoV-2 infection in our patients between these subtypes [46–48].

COVID-19 infected patients with poor outcome have been associated with lower levels of total cholesterol, HDL and LDL performed during the infectious period [49, 50]. In addition to ACE2 receptor, lipoprotein receptors could be another route of entry of SARS-CoV-2 into the host cell. The HDL cholesterol has been inversely correlated with the severity of the disease and was suggested as a potential prognostic blood biomarker [49–52]. HDL can facilitate a possible route of SARS-CoV-2 entry into the host cell via the SR-B1 receptor. Pretreatment of HEK-293T cells with a potent SR-B1 antagonist ITX5061 strongly inhibited the entry of SARS-CoV-2 into host cells [53]. In our study, levels of total cholesterol, HDL and LDL were not associated with the outcome since almost all patients were not assessed in the acute phase of COVID-19.

This study had some limitations. The retrospective character of the evaluation was based primarily on the serological diagnosis although the predictive value of the assay used would be up to 95%. We also did not assess the characteristics of exposure, once the objective of the study was not to determine the risk of sickness, but the prevalence of SARS-COV-2 infection in CGL patients, their clinical and metabolic characteristics and their association with outcomes.

We expected an increased risk of COVID-19 contamination once the lockdown was not carried out all over the period of the study, and we have a young and economically active population. However, a possible explanation is that most of these patients were jobless.

Also, the small number of patients evaluated may cause some bias. Nonetheless, this is one of the largest series of this rare disease in Brazil and the first study in this field.

## Conclusion

In the present study, we described for the first time a high prevalence of SARS-CoV-2 infection in CGL patients with a good outcome in all of them. These findings suggest that at least young CGL patients infected by SARS-COV-2 are not at higher risk of poor outcome, despite of known severe metabolic comorbidities.

## Data Availability

The datasets used and/or analyzed during the current study are not publicly available due risk that participants might be identifiable is considered non-negligible (indirect identifiers: age, sex, rare disease anthropometry measures, small denominator and numerators) but are available from the corresponding author on reasonable request.

## Abbreviations

A1c: Glycohemoglobin A1c
ACE2: Angiotensin converting enzyme 2
BRAZLIPO: Brazilian Group for the Study of Inherited and Acquired Lipodystrophies
CAD: Coronary artery disease
CGL: Congenital generalized lipodystrophy
CKD: Chronic kidney disease
COVID-19: Coronavírusdisease-2019
DM: Diabetes mellitus
F: Feminine
HCoV: Human coronavirus
HDL: High-density lipoprotein
HPLC: High-performance liquid chromatography
LDL: Low-density lipoprotein
M: Male
MTF: Metformin
Non-HDL: Non-high-density lipoprotein cholesterol
RAAS: Renin angiotensin aldosterone system
RT-PCR: Real*-*time polymerase chain reaction
SAH: Systemic arterial hypertension
SARS-COV-2 −: Non-reactive serologic tests for SARS-CoV-2 infection
SARS-COV-2+: Reactive serologic tests for SARS-CoV-2 infection
SARS-CoV-2: Severe acute respiratory syndrome coronavirus 2
TC: Total cholesterol
TG: Triglycerides

## References

[1] Li Q, Guan X, Wu P, Wang X, Zhou L, Tong Y (2020). Early transmission dynamics in Wuhan, China, of novel coronavirus-infected pneumonia. N Engl J Med.

[2] Wu F, Zhao S, Yu B, Chen YM, Wang W, Song ZG (2020). A new coronavirus associated with human respiratory disease in China. Nature.

[3] Zhou P, Lou Yang X, Wang XG, Hu B, Zhang L, Zhang W (2020). A pneumonia outbreak associated with a new coronavirus of probable bat origin. Nature.

[4] Ceará. Decreto nº 33574, de 5 de maio de 2020 de 2020. Institui, no município de Fortaleza, a política de isolamento social rígido como medida de enfrentamento à COVID–19, e dá outras providências. Fortaleza: Governo do Estado do Ceará; 2020 [cited 2021 Mar 9]. [4 p]. Available from: https://coronavirus.ceara.gov.br/project/decretos-estadual-e-municipal-instituem-politica-de-isolamento-rigido-a-partir-de-08-de-maio/

[5] Pan American Health Organization. World Health Organization. Epidemiological Update: Corona virus disease (COVID-19). Washington, D.C.: PAHO/WHO; 2020 [cited 2021 Mar 9]. [15 p]. Available from: https://www.paho.org/en/documents/epidemiological-update-coronavirus-disease-covid-19-23-june-2020

[6] Agarwal S, Schechter C, Southern W, Crandall JP, Tomer Y (2020). Preadmission diabetes-specific risk factors for mortality in hospitalizedpatients withdiabetes and coronavirus disease 2019. Diabetes Care.

[7] Holman N, Knighton P, Kar P, O’Keefe J, Curley M, Weaver A (2020). Risk factors for COVID-19-related mortality in people with type 1 and type 2 diabetes in England: a population-based cohort study. Lancet Diabetes Endocrinol.

[8] Wu J, Zhang J, Sun X, Wang L, Xu Y, Zhang Y (2020). Influence of diabetes mellitus on the severity and fatality of SARS-CoV-2 (COVID-19) infection. Diabetes Obes Metab.

[9] Gao F, Zheng KI, Wang XB, Sun QF, Pan KH, Wang TY (2020). Obesity is a risk factor for greater COVID-19 severity. Diabetes Care.

[10] Qingxian C, Fengjuan C, Fang L, Xiaohui L, Tao W, Qikai W (2020). Obesity and COVID-19 severity in a designated hospital in Shenzhen China. SSRN Electron J.

[11] Coppelli A, Giannarelli R, Aragona M, Penno G, Falcone M, Tiseo G (2020). Hyperglycemia at hospital admission is associated with severity of the prognosis in patients hospitalized for COVID-19: The pisa COVID-19 study. Diabetes Care.

[12] Selvin E, Juraschek SP (2020). Diabetes epidemiology in the covid-19 pandemic. Diabetes Care.

[13] Wang S, Ma P, Zhang S, Song S, Wang Z, Ma Y (2020). Fasting blood glucose at admission is an independent predictor for 28-day mortality in patients with COVID-19 without previous diagnosis of diabetes: a multi-centre retrospective study. Diabetologia.

[14] The Lancet Diabetes and Endocrinology (2020). COVID-19 and diabetes: a co-conspiracy?. Lancet Diabetes Endocrinol.

[15] Vamvini M, Lioutas VA, Middelbeek RJW (2020). Characteristics and diabetes control in adults with type 1 diabetes admitted with covid-19 infection. Diabetes Care.

[16] Apicella M, Campopiano MC, Mantuano M, Mazoni L, Coppelli A, Del Prato S (2020). COVID-19 in people with diabetes: understanding the reasons for worse outcomes. Lancet Diabetes Endocrinol.

[17] Bello-Chavolla OY, Bahena-López JP, Antonio-Villa NE, Vargas-Vázquez A, González-Díaz A, Márquez-Salinas A (2020). Predicting mortality due to SARS-CoV-2: a mechanistic score relating obesity and diabetes to COVID-19 outcomes in Mexico. J Clin Endocrinol Metab.

[18] Riddle MC (2020). Diabetes and covid-19: moving from news to knowledge and a glucose hypothesis. Diabetes Care.

[19] Narula S, Yusuf S, Chong M, Ramasundarahettige C, Rangarajan S, Bangdiwala SI (2020). Plasma ACE2 and risk of death or cardiometabolic diseases: a case-cohort analysis. Lancet.

[20] Soldo J, Heni M, Königsrainer A, Häring HU, Birkenfeld AL, Peter A (2020). Increased hepatic ace2 expression in nafl and diabetes-a risk for covid-19 patients?. Diabetes Care.

[21] Lampasona V, Secchi M, Scavini M, Bazzigaluppi E, Brigatti C, Marzinotto I (2020). Antibody response to multiple antigens of SARS-CoV-2 in patients with diabetes: an observational cohort study. Diabetologia.

[22] Chiquette E, Oral EA, Garg A, Araújo-Vilar D, Dhankhar P (2017). Diabetes, metabolic syndrome and obesity: targets and therapy dovepress estimating the prevalence of generalized and partial lipodystrophy: findings and challenges. Diabetes Metab Syndr Obes Targets Ther.

[23] Batista L, Medeiros A, Kristina V, Dantas C, Sales A, Sarmento C (2017). High prevalence of Berardinelli-Seip congenital Lipodystrophy in Rio Grande do Norte State Northeast Brazil. Diabetol Metab Syndr.

[24] Ponte CMM, Fernandes VO, Gurgel MHC, Vasconcelos ITGF, de Karbage LBA, Liberato CBR (2018). Early commitment of cardiovascular autonomic modulation in Brazilian patients with congenital generalized lipodystrophy. BMC Cardiovasc Disord.

[25] Liberato CBR, da Olegario C, Fernandes VO, Montenegro AP, da Lima CP, de Batista LA (2020). Early left ventricular systolic dysfunction detected by two-dimensional speckle-tracking echocardiography in young patients with congenital generalized lipodystrophy. Diabetes Metab Syndr Obes Targets Ther.

[26] Montenegro MR, da Lima CP, Fernandes VO, Pinheiro DP, de Moraes MEA, de MoraesFilho M (2020). Leu124Serfs * 26, a novel AGPAT2 mutation in congenital generalized lipodystrophy with early cardiovascular complications. Diabetol Metab Syndr.

[27] Montenegro RM, Fernandes VO, Saboia JGP, Montenegro APDR, Lima JG (2019). Type 2 congenital generalized lipodystrophy: the diagnosis is in your hands. J Pediatr.

[28] Patni N, Garg A (2015). Congenital generalized lipodystrophies—new insights into metabolic dysfunction. Nature Rev Endocrinol.

[29] Lima JG, Nobrega LHC, Lima NN, dos Santos MCF, Silva PHD, Maria de Fatima PB (2018). Causes of death in patients with Berardinelli-Seip congenital generalized lipodystrophy. PLoS One.

[30] Patni N, Garg A (2015). Congenital generalized lipodystrophies - New insights into metabolic dysfunction. Nature Rev Endocrinol.

[31] Roche Diagnostics International. Elecsys® Anti-SARS-CoV-2: Immunoassay for the qualitative detection of antibodies against SARS-CoV-2. Switzerland; 2020 [cited 2021 Mar 9]. Available from: https://diagnostics.roche.com/global/en/products/params/elecsys-anti-sars-cov-2.html

[32] Ministério da Saúde (BR), Instituto de Tecnologia em imunobiológicos—Bio-Manguinhos, FIOCRUZ. KIT MOLECULAR SARS-CoV2 (E)—Bio-Manguinhos. Rio do Janeiro; 2020 [cited 2021 Mar 9]. [9 p]. Available from: https://www.bio.fiocruz.br/images/molec-sars-cov2-e-96r-04-05-2020-lotes-11ao18.pdf

[33] Departamento Científico de Endocrinologia SBP. Dislipidemia na criança e no adolescente—Orientações para o pediatra. Guia Prático Atualização SBP. 2020;8(Maio):1–13

[34] Prefeitura Municipal de Fortaleza. Secretaria Municipal da Saúde. Soroprevalênca e estimativa de circulação viral do Coronavírus em Fortaleza: 2ª rodada. Dados preliminares. Agosto/2020. Fortaleza; 2020 [cited 2021 Mar 9]. [33 p]. Available from: https://www.fortaleza.ce.gov.br/images/0001/05_08_2020_COVID_INQU%C3%89RITO_05082020_Final.pdf

[35] Centers for disease control and prevention. Overview of testing for SARS-CoV-2 (COVID-19). Atlanta; 2020 [cited 2021 Mar. 9]. Available from: https://www.cdc.gov/coronavirus/2019-ncov/hcp/testing-overview.html

[36] Figliozzi S, Masci PG, Ahmadi N, Tondi L, Koutli E, Aimo A (2020). Predictors of adverse prognosis in COVID-19: a systematic review and meta-analysis. Eur J Clin Invest.

[37] Shah H, Khan MSH, Dhurandhar NV, Hegde V (2020). The triumvirate: why hypertension, obesity, and diabetes are risk factors for adverse effects in patients with COVID-19. Acta Diabetol.

[38] Sandooja R, Vura N, Morocco M (2020). Heightened ACE activity and unfavorable consequences in COVID-19 diabetic subjects. Int J Endocrinol.

[39] Cristelo C, Azevedo C, Marques JM, Nunes R, Sarmento B (2020). SARS-CoV-2 and diabetes: new challenges for the disease. Diabetes Res Clin Pract..

[40] Obukhov AG, Stevens BR, Prasad R, Calzi SL, Boulton ME, Raizada MK (2020). Sars-cov-2 infections and ace2: clinical outcomes linked with increased morbidity and mortality in individuals with diabetes. Diabetes.

[41] Li Y, Xu Q, Ma L, Wu D, Gao J, Chen G (2020). Systematic profiling of ACE2 expression in diverse physiological and pathological conditions for COVID-19/SARS-CoV-2. J Cell Mol Med.

[42] Kruglikov IL, Shah M, Scherer PE (2020). Obesity and diabetes as comorbidities for COVID-19: underlying mechanisms and the role of viral–bacterial interactions. elife.

[43] Paz-Filho G, Mastronardi C, Franco CB, Wang KB, Wong M-L, Licinio J (2012). Leptin: molecular mechanisms, systemic pro-inflammatory effects, and clinical implications. Arq Bras Endocrinol Metabol.

[44] Ludvigsson JF (2020). Systematic review of COVID-19 in children shows milder cases and a better prognosis than adults. Acta Paediatr.

[45] Mcmichael A, Simon AK, Hollander GA (2015). Evolution of the immune system in humans from infancy to old age. R Soc B Proc.

[46] Lima JG, Helena Nobrega LC, de NobregaLima N, do Goretti NSM, Baracho MF, Jeronimo MBS (2016). Clinical and laboratory data of a large series of patients with congenital generalized lipodystrophy. Diabetol Metab Syndr.

[47] Agarwal AK, Simha V, Oral EA, Moran SA, Gorden P, O’Rahilly S (2003). Phenotypic and genetic heterogeneity in congenital generalized lipodystrophy. J Clin Endocrinol Metab.

[48] Yang K, Sugii W, Han S (2014). Towards a mechanistic understanding of lipodystrophy and seipin functions. Biosci Rep.

[49] Hu X, Chen D, Wu L, He G, Ye W (2020). Low serum cholesterol level among patients with COVID-19 infection in Wenzhou, China. SSRN Electron J..

[50] Wei X, Zeng W, Su J, Wan H, Yu X, Cao X (2020). Hypolipidemia is associated with the severity of COVID-19. J Clin Lipidol.

[51] Hu X, Chen D, Wu L, He G, Ye W (2020). Declined serum high density lipoprotein cholesterol is associated with the severity of COVID-19 infection. Clin Chim Acta.

[52] Ding X, Zhang J, Liu L, Yuan X, Zang X, Lu F (2020). High-density lipoprotein cholesterol as a factor affecting virus clearance in covid-19 patients. Respir Med..

[53] Wei C, Wan L, Yan Q, Wang X, Zhang J, Zhang Y (2020). SARS-CoV-2 manipulates the SR-B1-mediated HDL uptake pathway for its entry. bioRxiv.

